# Flexion-Induced Cervical Cord Compression: Hirayama Disease

**DOI:** 10.4274/balkanmedj.galenos.2019.2019.1.16

**Published:** 2019-05-10

**Authors:** Sena Tolu, Fikret Aysal, Tuğrul Örmeci, İbrahim Ethem Kirez, Nurbanu Hindioğlu

**Affiliations:** 1 Department of Physical Medicine and Rehabilitation, Medipol University School of Medicine, İstanbul, Turkey; 2 Department of Neurology, Medipol University School of Medicine, İstanbul, Turkey; 3 Department of Radiology, Medipol University School of Medicine, İstanbul, Turkey

A 15-year-old man was presented with progressive weakness and amyotrophy of the right distal arm and hand muscles, which had been present for the last 3 months. The patient had no comorbid diseases and a history of cervical trauma. His family members had no neuromuscular disorders. A clinical examination showed weakness of the right interosseous muscles (grade 3/5 on the Medical Research Council scale), abduction of the right thumb, and extension of the right wrist and fingers II-V (Medical Research Council 4/5). On inspection, there was marked atrophy of the right first dorsal interosseous muscle and mild atrophy of the other intrinsic hand and flexion and extension muscles of the wrist. There was no fasciculation, sensory deficit, and pain, but tremulous movement of his fingers was observed. Deep tendon reflexes were intact. A neurologic examination of the left upper and lower extremities was normal, and no signs of pyramidal tract involvement were present. Babinski reflex and Hoffman’s sign were negative. Motor nerve conduction studies showed reduced amplitude of compound muscle action potential of the right ulnar nerve but normal parameters of the left ulnar and bilateral median nerves. No focal slowing or conduction block was found. Sensory nerve conduction studies were normal. An electromyographic examination found active denervation in the right C8-T1 and C7 innervated muscles (abductor pollicis brevis, first dorsal interosseus, abductor digiti minimi, extensor digitorum communis, flexor carpi radialis, and triceps muscles). A pure motor deficit affecting roots C7-T1 was diagnosed. Blood analyses were normal. Cervical vertebra magnetic resonance imaging performed in the neutral position was normal (Figure 1a, 1b). On the basis of clinical, electromyographic, and magnetic resonance imaging findings, we ruled out cervical cord pathologies, brachial plexopathy, multifocal motor neuropathy with conduction block, spinal muscular atrophy, and amyotrophic lateral sclerosis. On the suspicion of Hirayama disease, magnetic resonance imaging acquired in full flexion of the neck revealed dilation of the posterior internal vertebral plexus due to forward displacement of the posterior dura mater (Figure 1c-1f). The widening of the posterior epidural space disappeared in the neutral position. He was diagnosed to have Hirayama disease and treated conservatively. He was recommended to wear a cervical collar to minimize neck flexion. Written informed consent was obtained from the parent.

Hirayama disease is a rare self-limited condition characterized by asymmetric muscle weakness and amyotrophy of the distal upper limbs involving the C7-T1 myotomes. The pathologic findings implicate circulatory changes in the lower cervical cord, which causes ischemic changes in the anterior horn. Congestion of the venous plexus, abnormal drainage in the vertebral venous plexus, or malformation of the epidural vessels may be responsible (1,2). In this case, the diagnosis is guided mainly by electrophysiological and flexion magnetic resonance imaging findings.

## Figures and Tables

**Figure 1:**
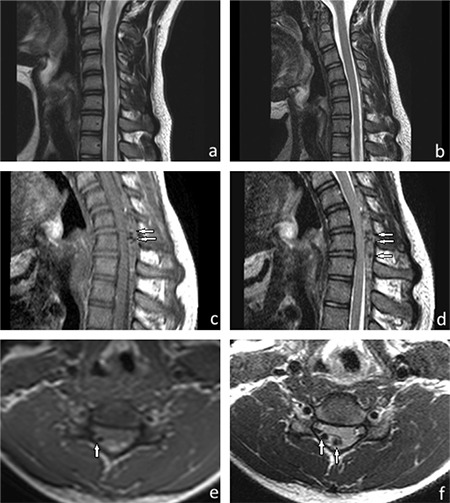
a-f. Hirayama disease. Normal magnetic resonance imaging findings on the sagittal T2-weighted images in a neutral position (a) and in slight flexion (b). However, dolichoectatic vascular structures (arrows) are seen on the sagittal T1-weighted image (c), sagittal T2-weighted image (d), axial T1-weighted image (e), and axial T2-weighted image in increased flexion (f). Prominent posterior epidural space and spinal cord compression are present between the C5 and D1 levels (c-f).
